# Generational conservation of composition and diversity of field-acquired midgut microbiota in *Anopheles gambiae* (*sensu lato*) during colonization in the laboratory

**DOI:** 10.1186/s13071-019-3287-0

**Published:** 2019-01-11

**Authors:** Jewelna Akorli, Philomena Asor Namaali, Godwin Williams Ametsi, Richardson Kwesi Egyirifa, Nana Adjoa Praba Pels

**Affiliations:** 1 West African Centre for Cell Biology of Infectious Pathogens, University of Ghana, P. O. Box LG 54, Legon, Accra Ghana; 2 Department of Parasitology, Noguchi Memorial Institute for Medical Research, University of Ghana, P. O. Box LG 581, Legon, Accra Ghana

**Keywords:** Midgut microbiota, *Anopheles gambiae* (*sensu lato*), Laboratory colonization, Field water, Breeding habitat

## Abstract

**Background:**

The gut microbiota is known to play a role in a mosquito vector’s life history, a subject of increasing research. Laboratory experiments are essential for such studies and require laboratory colonies. In this study, the conservation of field-obtained midgut microbiota was evaluated in laboratory-reared *Anopheles gambiae* (*s.l.*) mosquitoes continuously hatched in water from field breeding habitats.

**Methods:**

Pupae and late instars were obtained from the field and reared, and the emerged adults were blood-fed. The eggs obtained from them were hatched in either water from the field or in dechlorinated tap water. The mosquito colonies were maintained for 10 generations. Midguts of female adults from unfed F_0_ (emerging from field-caught pupae and larvae), F_5_ and F_10_ were dissected out and genomic DNA was extracted for *16S* metagenomic sequencing. The sequences were compared to investigate the diversity and bacterial compositional differences using ANCOM and correlation clustering methods.

**Results:**

Less than 10% of the bacterial families identified had differential relative abundances between generational groups and accounted for 46% of the variation observed. Although diversity reduced in F_10_ mosquitoes during laboratory colonization (Shannon-Weaver; *P*-value < 0.05), 50% of bacterial genera were conserved in those bred continuously in field-water compared to 38% in those bred in dechlorinated tap water.

**Conclusions:**

To our knowledge, this study is the first report on the assessment of gut bacterial community of mosquitoes during laboratory colonization and recommends the use of water from the natural breeding habitats if they are intended for microbiota research.

**Electronic supplementary material:**

The online version of this article (10.1186/s13071-019-3287-0) contains supplementary material, which is available to authorized users.

## Background

Mosquito vector-borne diseases are major health concerns, causing significant morbidity and mortality worldwide [1]. Although chemotherapy and the search for vaccines for these diseases have improved over the years, vector control remains a very important strategy. Faced with the challenges posed by resistance to insecticides, there have been increased efforts to find innovative methods of control through gaining a better understanding of factors that also influence both vector competence and capacity. One promising strategy involves exploring the use of midgut microbiota for transmission-blocking [2].

Bacteria inhabiting the midgut of mosquitoes contribute significantly to reducing the developmental capabilities of parasites that are ingested during a blood meal [3–5]. To date, a few bacterial species isolated from the midgut of wild-caught mosquitoes have been characterised for their roles in the life history of the mosquito (reviewed in [6]). Although such bacteria are isolated from natural populations, studies to investigate their functions make use of laboratory populations. Laboratory colonies of mosquitoes are usually well-adapted to laboratory conditions and are useful in performing experiments involving large numbers of mosquitoes. However, continuous laboratory maintenance of field-derived populations over several generations results in the loss of the native microbiota [7, 8], most likely due to changes in larval breeding water and other laboratory procedures. Therefore, results obtained by studies on microbiota might not be an accurate representation of what occurs in the wild [9].

In this study, we investigated the potential for breeding a population of ‘field’ mosquitoes in the laboratory with the aim of maintaining the midgut microbiota composition and diversity over generations. This could help establish large numbers of mosquitoes under laboratory conditions while conserving the natural midgut microbiota profile for further studies.

## Results

### Alpha diversity indices indicate similarity between treatment replicates

**Fig. 1:**
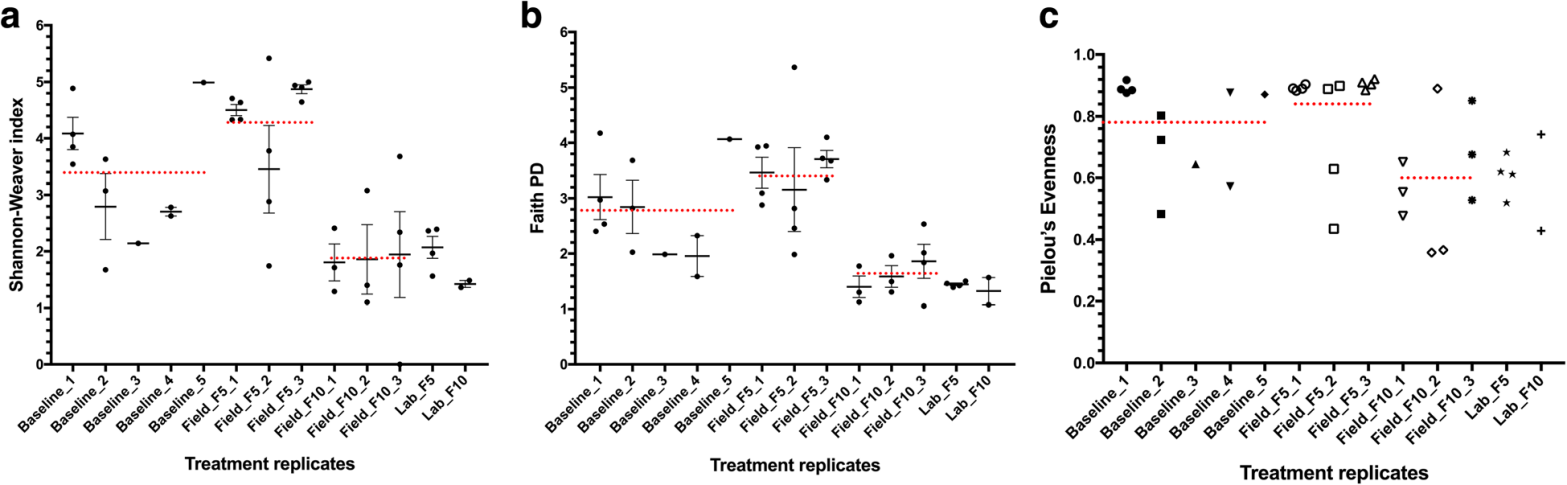
Shannon-Weaver (**a**), Faith’s phylogenetic piversity (PD) (**b**) and Pielou’s evenness indices (**c**) for samples. Black dots indicate index measure for each sample and error bars show standard error of the mean for each treatment replicate. Red dotted lines are the total average for all replicates for an experimental group

Taxon similarity and alpha diversity were compared between replicates for all treatment groups. This evaluation was done to assess possible differences between replicates, as sampling of mosquitoes and water samples from the field were performed over several days during the experimental period. Following rarefaction of sequences, some replicates were lost due to low sequence counts and resulted in only one representing the experimental treatment. Replicate comparisons were therefore impossible for such groups, i.e. Lab_F_5_ and Lab_F_10_. Shannon-Weaver and Faith’s phylogenetic diversity (PD) indices demonstrated similarity between replicates of the same experimental treatment (*P*-values > 0.05) (Table 1), although variations in means were observed (Fig. 1a, b). The evenness index gave no indication of a dominant species (index ≠ 0) (Fig. 1c), although an index of 0.36 (Fig. 1c; Field_F_10__2) may suggest a slight shift in taxon evenness in this replicate.

**Table 1:** Pairwise comparison (Kruskal-Wallis test) of diversity indices between sample replicates

Group 1	Group 2	Shannon-Weaver		Faith’s PD		Pielou’s evenness	
		H	Adjusted *P*-value	H	Adjusted *P*-value	H	Adjusted *P*-value
Baseline_1 (*n* = 4)	Baseline_2 (*n* = 3)	3.12	0.22	0.13	0.77	4.5	0.22
	Baseline_3 (*n* = 1)	2.00	0.31	2.00	0.30	2.0	0.44
	Baseline_4 (*n* = 2)	3.43	0.20	3.43	0.20	1.9	0.44
	Baseline_5 (*n* = 1)	2.00	0.31	0.50	0.56	2.0	0.44
Baseline_2 (*n* = 3)	Baseline_3 (*n* = 1)	0.20	0.74	1.80	0.30	0.2	0.75
	Baseline_4 (*n* = 2)	0.33	0.67	1.33	0.35	0.3	0.72
	Baseline_5 (*n* = 1)	1.80	0.33	1.80	0.30	1.8	0.44
Baseline_3 (*n* = 1)	Baseline_4 (*n* = 2)	1.50	0.37	0.00	1.00	0.0	1.00
	Baseline_5 (*n* = 1)	1.00	0.46	1.00	0.42	1.0	0.62
Baseline_4 (*n* = 2)	Baseline_5 (*n* = 1)	1.50	0.37	1.50	0.35	0.0	1.00
Field F_10__1 (*n* = 3)	Field_F10_2 (*n* = 3)	0.05	0.86	1.19	0.38	0.4	0.72
	Field_F10_3 (*n* = 4)	0.13	0.77	1.13	0.39	1.2	0.58
Field_F_10__2 (*n* = 3)	Field_F10_3 (*n* = 4)	0.13	0.77	0.50	0.56	0.4	0.72
Field_F_5__1 (*n* = 4)	Field_F5_2 (*n* = 4)	1.33	0.39	1.33	0.35	1.3	0.55
	Field_F5_3 (*n* = 4)	4.08	0.20	0.33	0.63	2.1	0.44
Field_F_5__2 (*n* = 4)	Field_F5_3 (*n* = 4)	1.33	0.39	1.33	0.35	3.0	0.32

### Number of observed features differed across generations

**Fig. 2:**
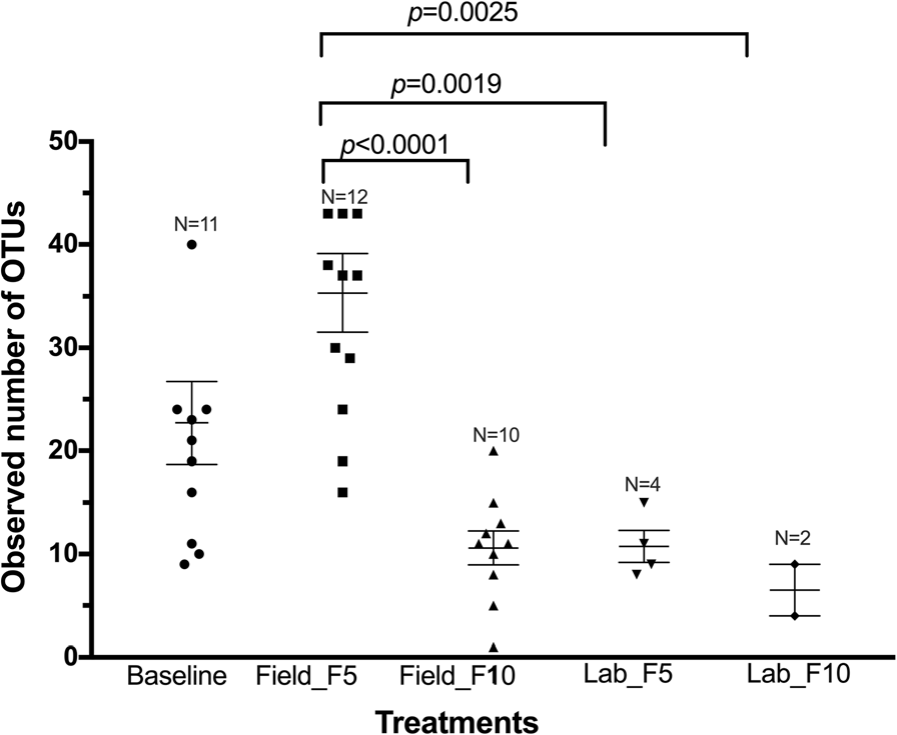
Comparison of observed OTUs between experimental treatments. Points are OTUs from samples (pool of 5 midguts) of each treatment. Error bars represent standard error of the mean

The diversity indices also gave a first-hand indication of differences between some experimental groups (Table 2). These were explored further by comparing features, operational taxonomic units (OTUs) and taxa classifications between treatments. The average number of OTUs ranged from as low as 6 in Lab_F_10_ to 35 in Field_F_5_ (Fig. 2). Interestingly, the average number of OTUs did not differ between baseline (F_0_) samples and any of the other experimental groups, likely due to the wide range of recorded points in some of the experimental groups and the reduced number of replicates. Field_F_5_ samples differed from all other groups except for the baseline (Fig. 2).

**Table 2:** Pairwise comparison of diversity indices between experimental groups

Group 1	Group 2	Shannon-Weaver	Faith’s PD	Pielou’s Evenness
Baseline (*n* = 11)	Field F5 (*n* = 12)	0.14	0.28	0.12
	Field F10 (*n* = 10)	0.02	0.005	0.07
	Lab F5 (*n* = 4)	0.12	0.01	0.16
	Lab F10 (*n* = 2)	0.04	0.04	0.25
Field F_5_ (*n* = 12)	Field F10 (*n* = 10)	<0.001	<0.001	0.001
	Lab F5 (*n* = 4)	0.008	0.0008	0.01
	Lab F10 (*n* = 2)	0.004	0.007	0.05
Field F_10_ (*n* = 10)	Lab F5 (*n* = 4)	0.81	0.67	1.0
	Lab F10 (*n* = 2)	0.50	0.64	0.92
Lab F_5_ (*n* = 4)	Lab F10 (*n* = 2)	0.45	0.90	0.94

Numbers are *P*-values following a Kruskal-Wallis test corrected for multiple comparisons by controlling false discovery rate

**Fig. 3:**
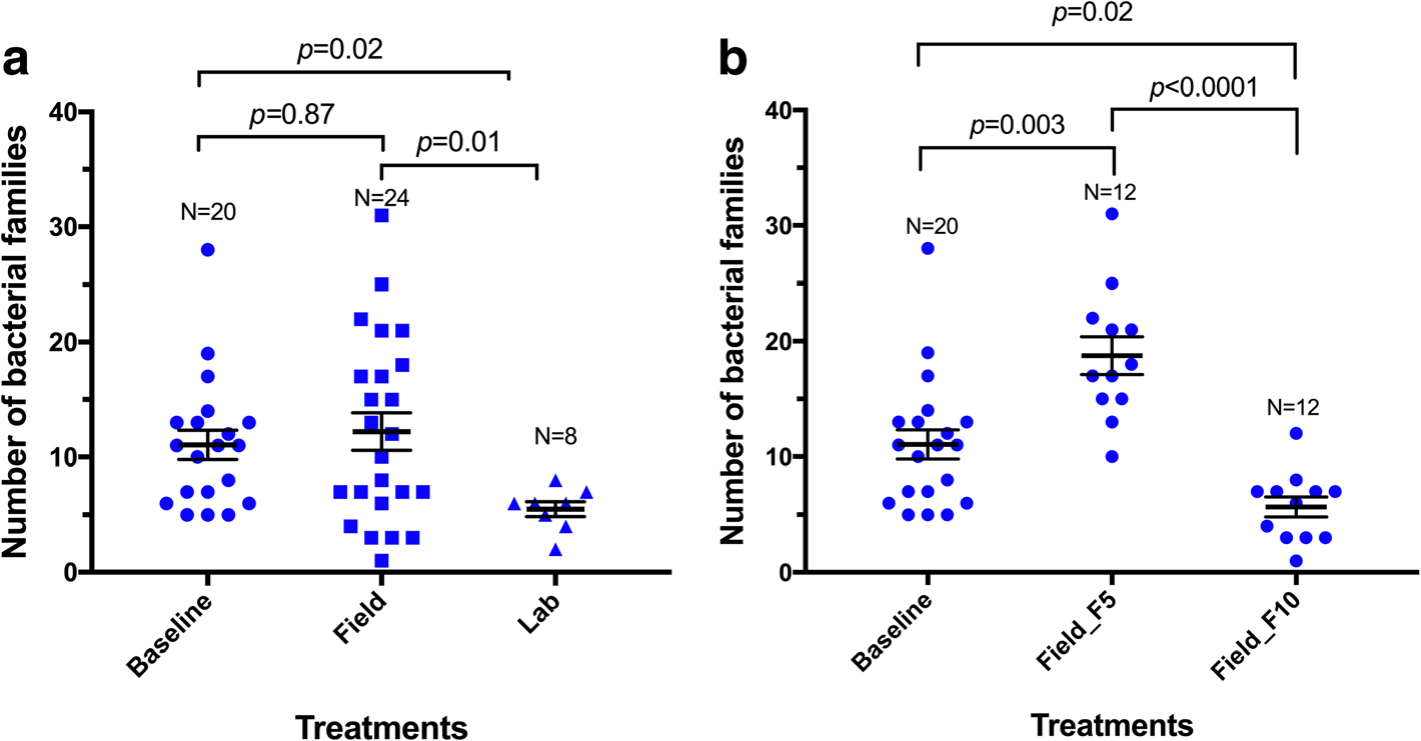
Comparison of 99 taxonomically identified bacterial families between experimental groups. Each point represents a sample (pool of 5 midguts). **a** Comparison between the baseline and all field and lab water bred samples. **b** Comparison of baseline and generational groups of field-water-bred mosquitoes. Error bars represent standard error of the mean

To be able to understand the source of the differences detected, the sequences were analysed based on bacterial families. In total, 99 families were taxonomically identified ranging from 1 to 31 per sample. The average number of bacterial families in mosquitoes reared in dechlorinated water was significantly lower than in field-water-reared (5 *vs* 12; Mann-Whitney U-test: *U*_(30)_ = 45.5, *P* = 0.026) and baseline mosquitoes (5 *vs* 11; Mann-Whitney U-test: *U*_(26)_ = 27 *P* = 0.0052) (Fig. 3a).

### Differential compositional analyses

**Fig. 4:**
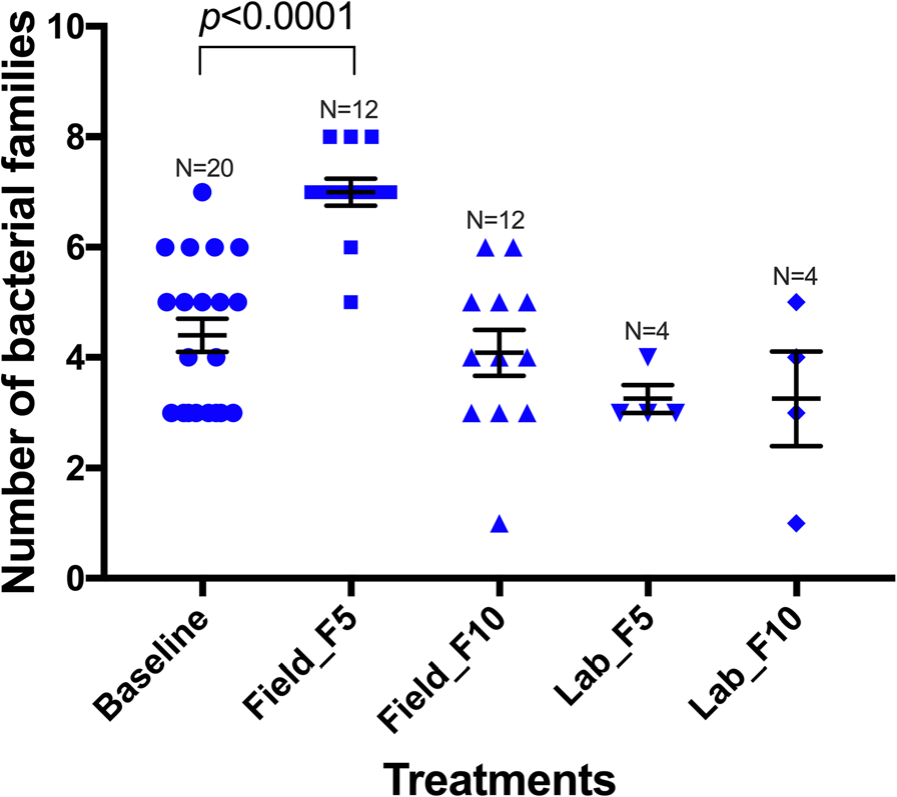
Comparison of 9 bacterial families (relative abundance ≥ 1% of sequences) between experimental groups. Points represent samples. Error bars represent standard error of the mean

We looked closer at the field water-bred mosquitoes to determine whether the observation made for Field_F_5_ during the OTU analyses persisted when analysed at the bacterial family level. Both groups of field-water-bred generations (F_5_ and F_10_) differed in family number from the baseline, the F_5_ showing a higher number than F_10_ in comparison to the baseline (Fig. 3b). However, about 91% (90 out of 99) of the families identified each represented < 1% of the total number of analysed sequences. Re-analysis based on the 9 families with relative abundance ≥ 1% revealed that Field_F_5_ remained higher than the baseline (Mann-Whitney U-test: *U*_(20)_ = 14, *P* < 0.0001), but Field_F_10_ and the dechlorinated water-reared mosquitoes were both similar to the baseline (*P* > 0.05) (Fig. 4).

**Fig. 5:**
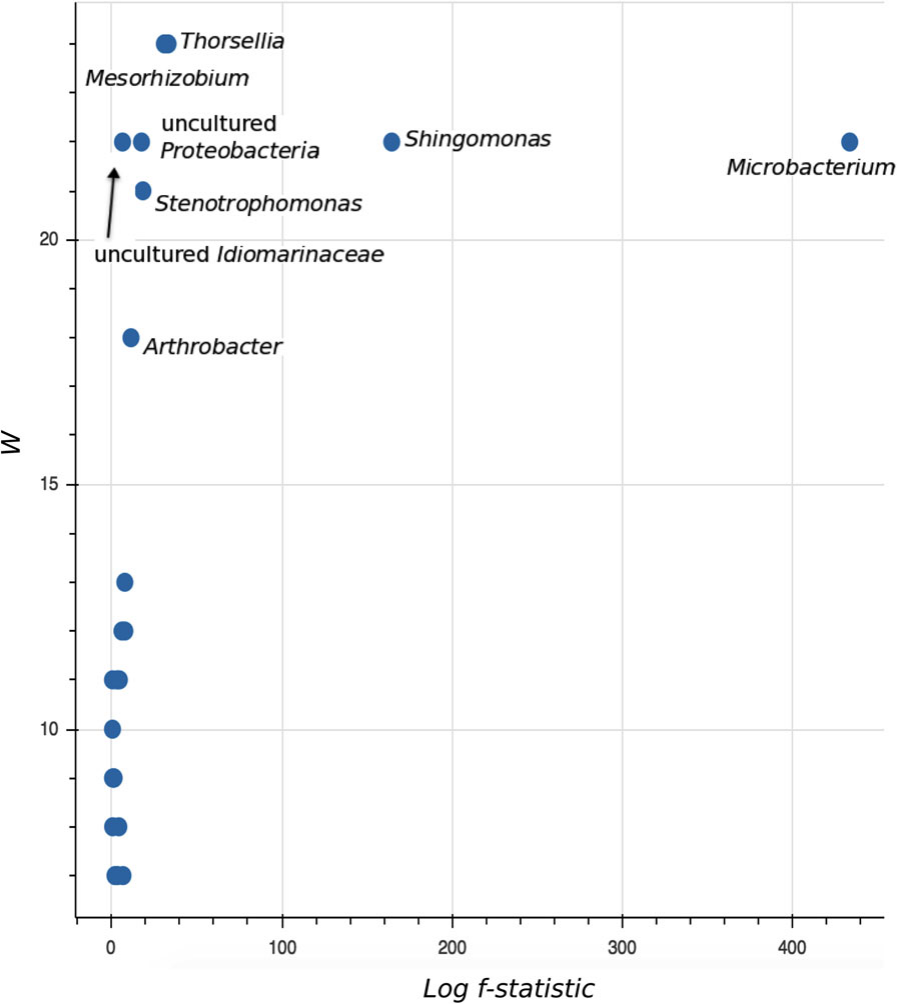
Volcano plot for the analysis of composition of microbiomes (ANCOM) test. Only significant bacterial taxa are labelled. Taxa on the top-left corner are distinct species but with small proportions, i.e. low f-score. Truly different taxa are depicted as one moves towards the far right (high W-statistic)

**Fig. 6:**
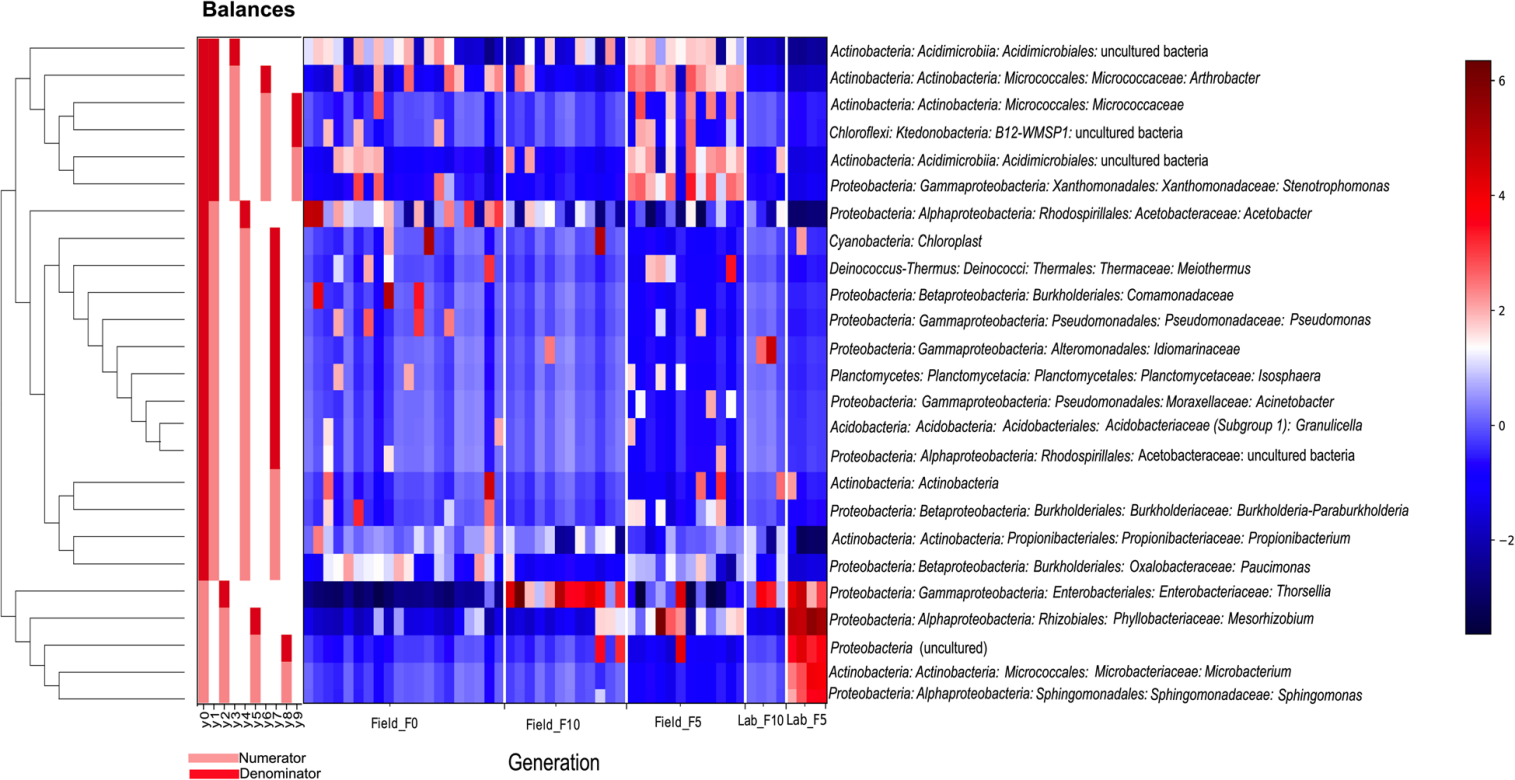
Dendogram of bacterial families resulting from unsupervised correlation clustering. Balances (*y0-y9*) are shown by pink (numerator) and red (denominator) vertical bars on the left side of the map, and are not related to the scale

The ANCOM results, which were based on bacterial genera, confirmed the contribution of a few taxa to the observed differences between groups of midguts (Fig. 5; Additional file 1: Table S1) and to 46% of the variance (Additional file 2: Table S3). Five (out of 8) of these significant taxa, *Thorsellia*, *Mesorhizobium*, *Microbacterium*, *Shingomonas* and some unspecified *Proteobacteria*, were more likely to cluster or co-occur (Fig. 6). The balance formed by these genera (*y0*_numerator) was more pronounced in samples bred in dechlorinated water for 5 generations (Lab_F_5_). The remaining 3 differential bacterial groups separately joined balances with other low relative abundance bacteria (Fig. 6). Four balances (*y1*, *y2*, *y5* and *y9*) significantly demonstrated which bacterial taxa contributed to the differences in Field_F_5_ and other samples bred in field water (Field_F_0_ and F_10_) (Additional file 2: Table S4). Notable among these are *Micrococcaceae* (genus *Arthrobacter*), *Xanthomonadaceae* (genus *Stenotrophomonas*), *Enterobacteriaceae* (genus *Thorsellia*) and *Phyllobacteriaceae* (genus *Mesorhizobium*) which significantly increased, while *Acetobacteraceae* (genus *Acetobacter*) and *Pseudomonadaceae* (genus *Pseudomonas*) decreased in Field_F_5_ compared to Field_F_0_ (baseline) (Fig. 6). Following 10 generations of laboratory maintenance, 50 and 38% of bacterial genera were conserved (maintained as present or absent) in field-water and dechlorinated tap water-bred mosquitoes, respectively (Additional file 1: Table S2).

## Discussion

Laboratory colonization of mosquitoes is useful for producing large numbers of samples for experiments on various aspects of mosquito life history, and selection of specific traits. However, for microbiota studies, the bacterial communities found in wild mosquitoes could be lost after a few generations, thus creating a discordance which limits the usefulness of laboratory colonies in efforts to understand the roles of microbiota [9]. The present study demonstrates that conservation of midgut microbiota can be achieved using water from breeding sites in the laboratory maintenance of mosquitoes. Relative bacterial abundances varied between generations of colonies continuously bred in field-water, while still maintaining significant field-derived taxa. However, dechlorinated water, as used in standard insectary procedures for egg hatching, resulted in a significant decrease in the relative abundance of many bacterial taxa while selectively keeping a few at high abundance.

Bacteria in the larval environment form a major part of the mosquito midgut through its aquatic developmental stages to emerged adults [10–12]. Breeding water is, however, dynamic with its bacterial community differing at different surface layers [13], and with abiotic and biotic effects such as contamination [14] and seasonal variations [15]. The laboratory environment is more controlled, presenting with less effects from natural external sources. Nevertheless, various factors in the laboratory may also cause changes in larval breeding water during mosquito maintenance. Such environmental variations could result in same mosquito species maintained in different insectaries having different distinct microbial communities. Further investigation on the influence of variations in laboratory environment on changes in microbiota could be important in understanding both the effect of laboratory conditions in shaping the midgut bacteria of colonized mosquitoes, and how this contributes to potential differences in experimental results between laboratories. With 10 generations of breeding field-caught mosquitoes in field-water under laboratory conditions, approximately 50% of the ‘natural’ microbiota was conserved compared to 38% in mosquitoes reared using dechlorinated tap water. The study did not show a significant difference in these percentages, likely due to small sample size.

Continuous breeding in field water may help replace some bacteria and introduce new ones that have not already been observed in the founding population, as observed in the unexpected increase in the number of bacterial families in the fifth generation of field-bred mosquitoes. Other bacteria may also be consistently lost once under laboratory conditions (Additional file 1: Table S2). The field-water returned to the laboratory at each collection point was not tested for bacteria in this study, but the dynamics of this environment to both natural biotic and abiotic factors could explain the variations observed from one field group to another.

The use of dechlorinated tap water, which is a standard practice in mosquito insectaries, poses an initial bottleneck for mosquito colonization as was observed in our study. Chlorine is an effective bacteria-inactivating and killing agent [16, 17]. This effect resulted in a small number of replicates in our tap-water reared as compared to field-water samples. That notwithstanding, these samples were able to demonstrate the reduction of bacterial families and relative abundance in samples brought into the laboratory after ten generations, consistent with reports of loss of bacterial populations in laboratory colonized mosquitoes [7, 8].

The use of balances on our dataset enabled the identification of major taxa whose relative abundances were most significant in explaining compositional variations. Most notable among these are the four taxonomically identified genera that formed part of the largest balance (*y0*): *Thorsellia*, *Mesorhizobium*, *Microbacterium* and *Sphingomonas*. These co-occurring bacteria became most pronounced in tap-water reared samples despite rarely occurring at baseline. This is indicative of these bacterial taxa having a proliferation advantage when many others have been lost due to some selective pressure [18]. The great extent of bacteria loss during laboratory rearing results in distinct profiles dominated by few species compared to field-caught mosquitoes [19], as demonstrated in our study. Again, the small number of tap-water reared samples limited the extent to which this could be observed. Despite this *Microbacterium* and *Sphingomonas* showed distinctly in our tap-water reared mosquitoes and demonstrated significant difference in abundance. Both bacterial taxa have been identified in field-caught and lab-reared *Anopheles* species [20, 21].

The persistence and increase in the incidence of *Thorsellia* in laboratory colonized mosquitoes cannot be ignored. This bacterial genus has been isolated from the midguts of some *Anopheles* malaria vectors [8, 22, 23] and *Culex* mosquitoes [24]. They are known to increase growth in blood medium [23], hence could potentially be involved in blood digestion in mosquitoes. Besides the midgut, *Thorsellia* spp. are also found to inhabit the reproductive tracts of both sexes of *Anopheles gambiae* and *An. coluzzii* [25], necessitating further investigations on the functions of these bacteria in malaria mosquitoes.

## Conclusions

We have demonstrated the conservation of field-derived bacterial community in mosquitoes maintained under laboratory conditions for ten generations by field-water replacement. This study also confirms the loss of microbial profile when mosquitoes are bred in tap water, which is a standard laboratory practice. The ability to breed large populations of mosquitoes for controlled experiments could help provide answers to the contribution of microbiota to vector competencies to disease transmission.

## Methods

### Mosquito samples and experimental set-up

Late (3rd and 4th) instars and pupae of *An. gambiae* (*s.l*.) were sampled together with water from a breeding site in peri-urban Accra and transported to the laboratory in plastic containers. Three to four batches of mosquito samples were collected from the field within 3–4 days of each other. The pupae were separated into cups and placed in cages with no source of sugar meal for the emerging adults. The larvae were transferred into larval trays and maintained without adding larval food for a maximum of 3 days under standard insectary conditions. Remaining larvae were discarded. Emerging pupae were collected from the larval trays each day and transferred into cages wiped with 70% ethanol. For each batch of field collection, 30 one-day-old non-sugar-fed females were stored at -20 °C until needed for midgut dissection. The remaining adult mosquitoes in the cage were offered 10% glucose through cotton balls for 4–5 days and blood-fed.

F_1_ eggs collected from each batch of field collection were divided into two groups and placed in larval trays for the experiments. One group was placed in a tray containing field water (collected the previous day and sieved through a cloth to ensure mosquito eggs and early stage larvae were removed before use) and the other in dechlorinated tap water (chlorinated tap water left standing for at least 24 h). To standardize the set-up and prevent overcrowding, the egg to water ratio of 1 egg to 20 ml was maintained. Yeast and larval food were added to all trays daily. The water level was replenished with one-third of water (as appropriate for each tray) every other day to prevent drying. The larvae were observed daily, and the dead were removed. Emerging adult mosquitoes were maintained through generations as described above on sugar and blood. At 5 and 10 generations, emerged 1-day-old unfed female mosquitoes were sub-sampled (~30 female adults) for midgut dissections.

### Midgut preparation and sequencing

Midguts were dissected from stored female adult mosquitoes sampled from F_0_ (baseline), F_5_ and F_10_ generations. A sample contained 5 dissected mosquito midguts, and for each treatment replicate a maximum of 4 samples (i.e. 20 midguts) was obtained. The total number of samples obtained for each experimental group (F_0_, Field_F_5_, Field_F_10_, Lab_F_5_, Lab_F_10_) ranged between 4 and 20 (Additional file 3: Table S5). Three sham samples were prepared during dissections as previously described [15]. Genomic DNA was extracted from each replicate and the 3 shams using QiaAmp Micro DNA kit (Qiagen, Hilden, Germany) according to manufacturer’s recommendations. The bacterial *16S* V1-3 region was sequenced for 55 DNA samples (Additional file 3: Table S5) on an Illumina MiSeq platform (Pretoria, South Africa) using the primers 27F (5'-AGA GTT TGA TCM TGG CTC AG-3') and 534R (5'-ATT ACC GCG GCT GCT GG-3').

Quality filtering, which included trimming the sequences to retain bases with > 20 Phred score and demultiplexing, was performed, and a total of 527,005 sequences were retrieved. The length of the retained sequences ranged between 35–295 bp. We performed further sequence filtering to remove very low sequence lengths, which could be problematic in downstream data analyses, such as taxonomic assignments [26]. A total of 513,720 sequences with lengths between 150–295 bp were extracted. The minimum and maximum sequence count per sample was 1276 and 36,171, respectively, with a mean of 9340.

### Screening sequences for potential ‘contamination’

Analyses of microbial data were performed using QIIME 2 (https://qiime2.org) following the “Moving Pictures” tutorials [27]. The paired end reads for all samples were imported into *qiime2* and the sequence summaries were visualized by using the *qiime demux* summarize plugin. The resulting *Interactive Quality Plot* was examined to truncate the sequences in both read directions at base positions where the read quality fell below the threshold of 20. Using this cut-off criterion resulted in pruning the forward and reverse sequences at 295 and 246 base positions, respectively, and produced a set of output representative sequences (32,504 in total). These were aligned and masked to remove highly variable positions and used to build a mid-point rooted tree for phylogenetic diversity analyses. The Naive Bayes classifier was trained on SILVA 128 [28, 29] 97% OTUs, with taxonomic reference set to extract and include the target sequence between the forward and reverse primer. Taxonomic classification was performed for representative sequences with *classify-sklearn* [30] in the *qiime2* feature-classifier plugin [31].

The resulting dataset was screened at the bacterial family level for possible ‘contamination’. To do this, the average relative abundance of bacterial families was calculated for the shams and those > 0.01 (Additional file 4: Tables S6, S7) were analysed for correlation with initial DNA concentration as previously described [15, 32]. No bacterial taxon was identified as a contaminant in our dataset.

### Analyses of experimental samples

The inclusion of the sham sequences could potentially influence downstream analyses, therefore a second dataset of 470,772 for the 52 test samples was created, which excluded the shams from the initial 513,720 sequences. These were taken through the processing and analyses pipeline as described above. The cut-off criterion for pruning the forward and reverse sequences changed to positions 291 and 247, respectively, and resulted in 52,312 sequences. Sequences designated as ‘Unidentified bacteria’ and ‘Unassigned’ were excluded from dataset used for the diversity and differential composition analyses.

Rarefaction for diversity analyses was performed at a sampling depth of 250 sequences per sample, which resulted in the loss of 8 samples with low sequence counts. The remaining 44 samples were analysed for taxon richness, evenness and diversity using the Faith’s phylogenetic diversity (PD), Pielou’s and Shannon-Weaver indices, and explored for observed operational taxonomic units (OTUs). The significance of alpha diversity was determined using Kruskal-Wallis test, accepting only adjusted *P*-values < 0.05 as significant.

Analysis of composition of microbiomes (ANCOM) was used to identify differential relative abundance of bacterial genera [33], and balance trees to evaluate changes (growth or decline) in microbial sub-communities between experimental groups [34]. As both methods of analysis are sensitive to less informative features (sequence grouping), the taxa frequency table was filtered to remove bacterial classifications with less than 10 reads and those observed in less than 3 samples in the study. To tolerate the zero frequencies of bacterial counts, a pseudo-count composition table was created by adding a count of 1 to every value. For ANCOM, the composition table was log-transformed and significance determined from f-scores and ‘W’ statistics. The f-score measures the strength of the difference of a feature between groups. A high score indicates the more likelihood that the null hypothesis (the average of the feature in all groups are the same) can be rejected. The ‘W’ statistics indicate the number of times a feature is detected to be significantly different across groups. Principal balances were built with unsupervised hierarchical clustering and isometric log ratio (ILR) transformation [35] to group features based on how frequently they co-occur. An ordinary least square regression model was fitted to the balances using the different generation of samples as the only predictor variable. Coefficient *P*-values were accepted at a stringent significance level of 0.01.

## Additional files

Additional file 1: **Table S1.** ANCOM results. **Table S2** Calculation of bacterial genera conservation in laboratory-colonized samples. (XLSX 17 kb)

Additional file 2: **Table S3.** Simplicial linear regression summary. **Table S4.** Regression coefficients and *P*-values. (XLSX 15 kb)

Additional file 3: **Table S5.** Details of 55 samples submitted for metagenomic sequencing. (XLSX 9 kb)

Additional file 4: **Table S6.** Relative abundance of bacterial families in shams. **Table S7.** Relative abundance of bacterial families in experimental samples. (XLSX 36 kb)

## Abbreviations

ANCOM: Analysis of Composition of Microbiomes
OTU: Operational Taxonomic Unit
